# Advances in the adsorption of heavy metal ions in water by UiO-66 composites

**DOI:** 10.3389/fchem.2023.1211989

**Published:** 2023-06-20

**Authors:** Yuanhang Lei, Jiangqin Xie, Wenxuan Quan, Qi Chen, Xingyu Long, Anping Wang

**Affiliations:** ^1^ School of Materials and Architectural Engineering, Guizhou Normal University, Guiyang, Guizhou, China; ^2^ Key Laboratory for Information System of Mountainous Area and Protection of Ecological Environment of Guizhou Province, Guizhou Normal University, Guiyang, Guizhou, China; ^3^ School of Chemistry and Materials Science, Guizhou Normal University, Guiyang, Guizhou, China

**Keywords:** metal-organic framework, composite material, adsorption, heavy metal ions, synthesis

## Abstract

The innovative adsorbents known as the Metal-organic Framework (MOFs) had a high specific surface area, various structural types, and good chemical stability. MOFs have been produced through hydrothermal, mechanochemical, microwave-assisted, gelation, and other synthesis methods, and the solvothermal process is one of them that researchers frequently utilize. The UiO materials have a more comprehensive application potential than different subtypes of MOFs among the numerous MOFs that have been synthesized. The synthesis of MOFs and their composites, as well as the adsorption characteristics of UiO materials in the adsorption of various heavy metal ions, have all been examined and summarized in this study.

## 1 Introduction

Metal-organic frameworks (MOFs) are crystalline materials with organic linkages bound to metal centers. They offer a new, promising class of adsorbents characterized by their substantial surface area, diverse high-quality structures, and chemical stability. Since their discovery in 1995 (Yaghi et al., 1995), the synthesis of more than 20,000 MOF compounds has been reported (Deng et al., 2012; Maurin et al., 2017), leading to their widespread utilization in the adsorption and catalytic industries. Among these, amino-functionalized MOFs, the UiO-66 type with zirconium as the central body, have emerged as potential candidates for heavy metal ion adsorption due to their acid and base resistance and exceptional structural stability.

Various preparation methods have been explored as the application of MOFs becomes increasingly prevalent. Throughout the manufacturing process, factors such as the coordination environment, coordination linkage, metal center ion, and chemical ligands significantly influence the structure of MOFs (Wang et al., 2013). Several reaction variables, including temperature, the molar ratio of metallic ions to organic ligands, solvent, pH of the reaction system, component concentration, and reaction time, have been identified as critical determinants of the resulting MOF structure and properties (Deng et al., 2015). MOFs’ design and control are more straightforward than traditional porous materials, as they can be synthesized under controlled and mild conditions, leading to materials with enhanced surface areas, permeabilities, heat resistance, and electrical characteristics (He et al., 2017; Huo et al., 2017).

**FIGURE 1:**
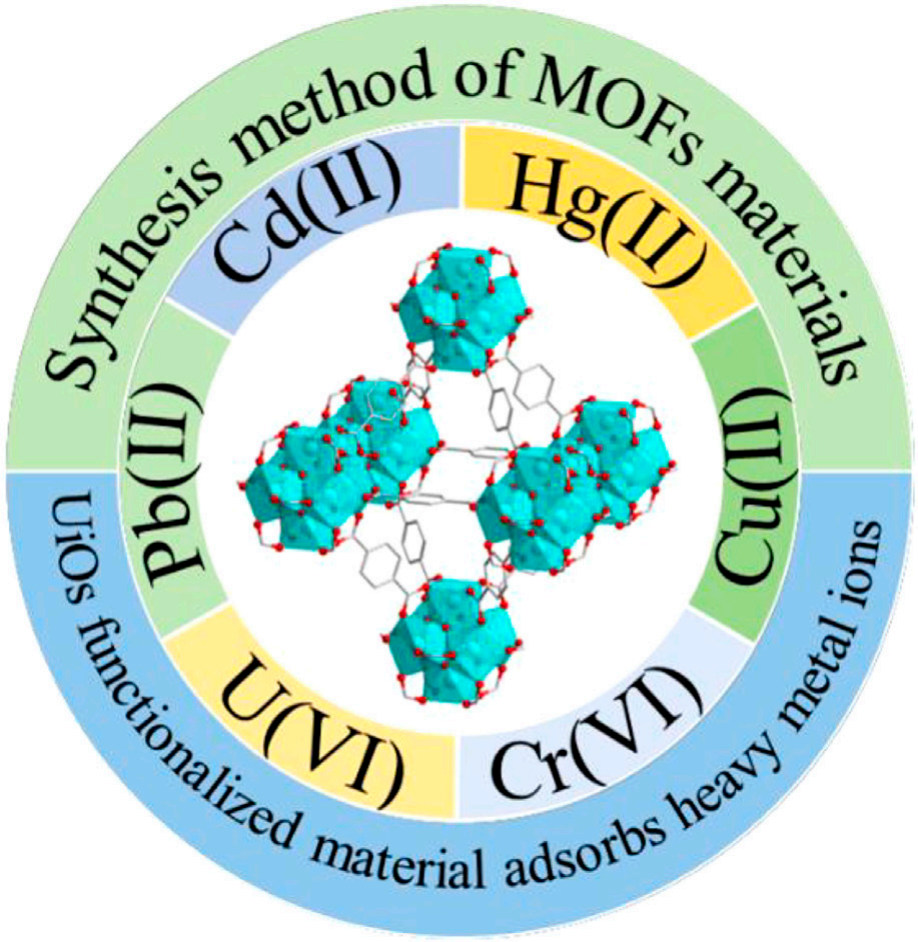
UiO-66 adsorption of heavy metal ions.

MOF materials offer versatility in synthesis methods and exhibit excellent adsorption properties for heavy metal ions, making them valuable in practical applications. Heavy metals pose severe environmental hazards due to their high toxicity and non-degradability, leading to detrimental effects on the central nervous system and accumulation in vital organs such as the brain and liver. Therefore, in this study, we present a comprehensive review of different synthetic methods for MOF synthesis and their composites, focusing on the application and future development of UiOs materials as adsorbents for the removal of heavy metal ions, including Pb(II), Cd(II), Cr(VI), and Hg(II) (Figure 1). This research aims to contribute to the advancement of efficient and sustainable strategies for heavy metal ion remediation.

**FIGURE 2:**
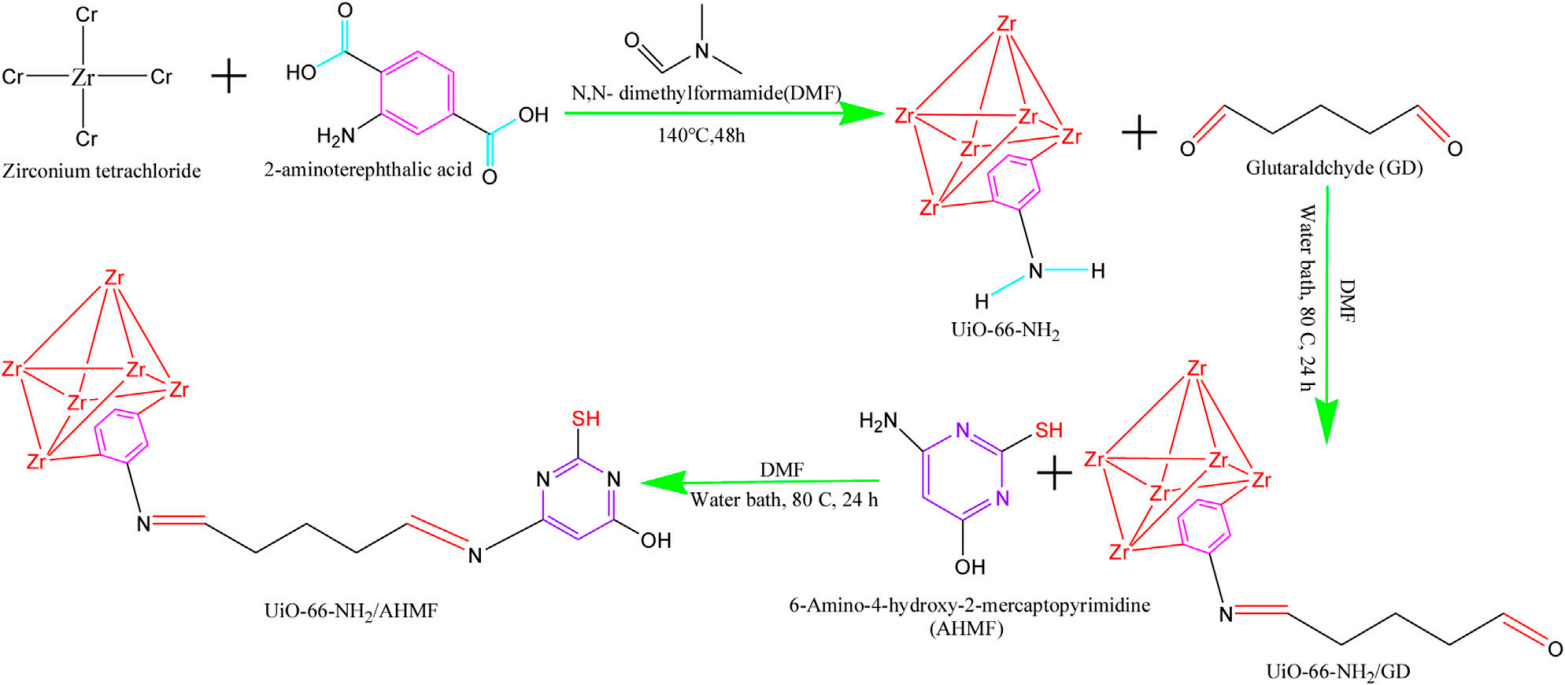
UiO-66-NH_2_/AHMP synthetic processes. (Figure 2 from Ref (Ruan et al., 2022). reproduced with permission of the author).

## 2 Synthesis method of MOFs materials

### 2.1 Solvothermal synthesis of MOFs

The solvothermal method has emerged as a preferred technique for synthesizing MOFs due to its ability to control product size, shape, and crystallinity precisely (Wang and Ho, 2016). This approach encloses a mixture of metal ions, organic ligands, conditioning agents, solvents, and other materials within a PTFE lining. Wang X. et al. (2018) employed a hydrothermal method to synthesize copper-based MOFs, abbreviated as Cu-MOFs. The study demonstrated the achievement of high crystallinity and a stable morphological structure, despite the time-consuming nature of the procedure and its relatively low yield (Wang et al., 2014).

### 2.2 Mechanochemical synthesis of MOFs

The mechanochemical or grind approach can produce MOFs through mechanical stirring or impacts between components (Wei et al., 2017). It is recognized as an environmentally friendly synthetic technique (Singh et al., 2017). This approach successfully reduces reaction times, eliminates the need for high temperatures, and minimizes or eliminates the use of organic solvents (Jing et al., 2006; Wang, 2013; Han et al., 2017; Kubota et al., 2019). Mechanochemical synthesis methods for MOF materials can be categorized into three groups: 1) Neat grinding (NG) (Pichon et al., 2006), where no solvent is utilized throughout the reaction process. 2) Liquid-assisted grinding (LAG) (Friščić et al., 2006; Friščić and Fábián, 2009) accelerates the mechanical chemical reaction by introducing a small amount of solvent, thereby enhancing reactant activity at the molecular level. 3) Ion-and-liquid aided grinding (ILAG) (Friščić et al., 2010), which expedites MOF synthesis by simultaneously employing a small amount of solvent and salt ions.

### 2.3 Synthesis of MOFs by microwave-assisted method

The microwave-assisted synthesis method offers a more efficient and straightforward alternative to the hydrothermal method for synthesizing Bulk-Al-PMOF. The hydrothermal method typically requires a lengthy reaction time of 16 h at 180°C. In contrast, the microwave method significantly shortens the synthesis time. Furthermore, the size of UiO-66 crystals grown using the microwave method was approximately 100 nm, four times smaller than the crystals produced using conventional heating, measuring around 400 nm (Li et al., 2014). This reduction in crystal size can be attributed to increased nucleation caused by the heating process, leading to the formation of smaller crystals with more nuclei (Li et al., 2014; Vakili et al., 2018).

In addition to crystal size reduction, the microwave method was employed in synthesizing UiO-66 (Taddei et al., 2015), resulting in an end product with a narrower pore-size distribution. Notably, the microwave technique substantially reduces synthesis time compared to conventional solvent heating, decreasing from 24 h to just 0.5 h.

### 2.4 Synthesis of MOFs by diffusion

The gel diffusion method involves mixing various materials for an extended period to facilitate the formation of MOF crystals through gel branching, wherein organic ligands are dispersed within the gel (Dhanya et al., 2013). Liquid-phase diffusion can be utilized by dissolving the central ion and organic ligand in an incompatible solvent. These solutions can be combined, and upon contact, MOF crystal products are generated (Xie et al., 2017). In the case of gas-phase diffusion, a volatile organic ligand solution serves as the solvent. MOFs can be synthesized by combining solutions of an organic ligand and core ion (Wu et al., 2016). The diffusion method is commonly employed for MOF synthesis, typically carried out over an extended period under mild reaction conditions (Wang F. X. et al., 2017). Shearer et al. successfully produced transparent UiO-66 using the diffusion approach (Shearer et al., 2013); however, the process required 2 weeks at 100°C. A simplified schematic illustrating the formation of MOFs via gas-phase diffusion is provided (Bian et al., 2018).

## 3 Heavy metal ion adsorption by UiOs

The 20th century witnessed a significant surge in industrial growth, necessitating substantial energy consumption. However, this growth has resulted in energy supply challenges, ecological damage, and environmental degradation (Li et al., 2021). Dealing toxic heavy metals and large quantities of contaminants into water severely threatens human life and other species. Heavy metal contamination remains at the top of the “blacklist” among various forms of water pollution due to its persistent nature and difficulty in containment and recovery. Addressing this issue requires effective wastewater treatment methods.

Several approaches are employed for the treatment of heavy metal pollution in wastewater, including chemical precipitation (Sarker et al., 2018), ion exchanges (Hashim et al., 2011), membrane separation (Chen et al., 2021a), evaporation (Chen et al., 2010), electro-coagulation (Bouhamed et al., 2012), and sorption (Al-Shannag et al., 2015; Chen et al., 2021b; Dong et al., 2021). Among these, the adsorption technique is widely recognized as the optimal choice for removing contaminants from water (Dinari and Tabatabaeian, 2018; Duman et al., 2020; Hoslett et al., 2020; Tabatabaeian et al., 2021). This technique is valued for its simplicity (Wu et al., 2018), efficiency (Liu et al., 2019), environmental friendliness (Chen et al., 2018), excellent removal efficacy (Zhang et al., 2017), and affordability (Wang et al., 2020).

### 3.1 Adsorption of Pb(II)

Lead (Pb) is a toxic and heavy metal that poses risks to human health and the environment. It is primarily generated through industrial wastewater treatment, battery disposal, and paint coating (Cechinel et al., 2014). Due to its non-biodegradable nature, Pb can accumulate in organisms, gradually infiltrating food chains and posing a threat to various species (Dong et al., 2010). Once collected in the human body, Pb exhibits extreme toxicity, affecting nearly all central organ systems. This can lead to conditions such as anemia, kidney disorders, cardiac disease, cerebral injury, cancers, endocrine disorders, and potentially fatal damage to the liver and reproductive functions (Charlet et al., 2012; Skipper et al., 2016).

In their study, Fu et al. (2019) achieved the efficient and targeted separation and removal of Pb(II) from water by converting the amino group in UiO-66-NH_2_ to a hydroxyl group using resorcinol formaldehyde, resulting in the synthesis of UiO-66-RSA. Under optimal conditions, UiO-66-RSA exhibited a maximum adsorption capacity of 189.8 mg/g for Pb(II), and even after five repeated use experiments, the adsorption rate only decreased by 3.7%.

### 3.2 Adsorption of Cr(VI)

Hexavalent chromium [Cr(VI)] is a widely used and highly hazardous heavy metal ion in various industries such as mining, tanning, metallurgy, and dyeing (Altundogan, 2005). Its exceptional water solubility and mobility contribute to the significant health risks it poses to humans, including genotoxic, mutagenic, teratogenic, and carcinogenic effects (Testa et al., 2004; Costa and Klein, 2006; Mohan and Pittman, 2006; Rapti et al., 2016; Zhang et al., 2018).

In their study, Fidelli et al. (2021) synthesized zirconium benzodicarboxylate (UiO-66) and zirconium benzodicarboxylate-NH_2_ (UiO-66-NH_2_) using mechanochemical methods for investigating Cr(VI) adsorption. The authors compared the adsorption characteristics of UiO-66 and UiO-66-NH_2_, synthesized through liquid-assisted grinding (LAG) and solution methods, under aqueous conditions. Four compounds were named UiO-66 (LAG), UiO-66-NH_2_ (LAG), UiO-66 (SOL), and UiO-66-NH_2_ (SOL). The results revealed that UiO-66-NH_2_ (LAG) exhibited a maximum Cr(VI) adsorption capacity of 36.6 ± 0.9 mg/g.

Shi et al. (Peng et al., 2021) developed a membrane adsorbent solution by coating a polyethersulfone (PES) membrane with a mixture of tannic acid, chitosan, and UiO-66 to remove Cr(VI) from water. The researchers varied the MOF loads and maintained a pH of 3.5 while fabricating the membrane-like adsorbent. The resulting adsorbent demonstrated excellent denseness, rapid separation, and high removal efficiency for Cr(VI) in water. Specifically, the addition of 30% chitosan loading enhanced the maximum removal rate of all three pollutants, resulting in a 99% removal rate in conjunction with the weight of UiO-66.

### 3.3 Adsorption of Cd(II)

Cd(II), a highly toxic metal in the environment, poses significant concerns for ecosystems and human health. It can cause lesions in various organs, particularly the kidneys (Flora et al., 2012). Furthermore, Cd(II) exposure has been linked to bone damage, cancer, tracheitis, chronic obstructive lung disease, eschar formation, and fibrillation (Ihsanullah.Abbas et al., 2016). Although several methods are available for Cd(II) removal, traditional sorbents like activated carbon often exhibit limited sorption capacities and lack adaptability (Kim et al., 2015; Lin et al., 2015). Therefore, the development of novel and efficient sorbents is of utmost importance.

In their study, Jamshidifard et al. (2019) synthesized UiO-66-NH_2_ using microwave heating and incorporated it into PAN chitosan nanofiber membranes. This composite material was utilized for both sorption and membrane filtration to remove Cd(II) from water. The results revealed that under optimal conditions, the adsorbent exhibited a maximum sorption capacity of 415.6 mg/g for Cd(II).

### 3.4 Adsorption of U(VI)

Uranium is a globally abundant resource and a significant fuel source. However, the mining and processing of uranium result in the generation of large quantities of uranium-containing wastewater. Without proper treatment, radioactive uranium nuclides can migrate through subsurface percolation and surface runoff, contaminating the ecosystem and wasting uranium resources (Peng et al., 2014; Xiao et al., 2015; Wang C. et al., 2018). The wastewater from uranium mines is complex in composition, with low uranium levels and the coexistence of various ions during practical production (Ren et al., 2015). To address the adsorption of U(VI), Zhang et al. (2022) employed amine oxime derivatives of UiO-66-(OH)2, which were chemically modified from UiO-66. These derivatives exhibited similar morphology to UiO-66(OH)2 but had significantly larger surface areas, more activated centers, and a higher affinity for binding U(VI). Yin et al. (Cheng et al., 2018) developed the porous material HP-UiO-66-35 by adjusting the dodecanoic inducer acid and reducing the particle size to 35 nm. This material demonstrated excellent adsorption performance.

To enhance the adsorption capabilities further, Wu et al. (2022) created g-C_3_N_4_/UiO-66 composites (CNUIO) by immobilizing UiO-66 onto a graphitic carbon nitride (g-C_3_N_4_) adsorbent surface. This composite overcomes the limitations of individual components by increasing specific surface areas, enriching surface functionality groups, and enhancing U(VI) adsorption capacity. CNUIO offers a practical and effective adsorbent for uranium water purification.

### 3.5 Adsorption of Hg(II)

Mercury (Hg) is considered to have the highest biological toxicity among non-biodegradable heavy metal ions. The World Health Organization and the European Union have set strict guidelines for Hg concentrations in clean water, with recommended limits below 1 *μ*g/mL and 1 *μ*g/L, respectively. To address this issue, Ruan et al. (2022) successfully developed a novel complex adsorbent called UiO-66-NH_2_/AHMP to extract Hg(II) from aqueous solutions. The morphological features of the adsorbents before and after modification were analyzed using FE-SEM. The similar sizes and morphologies observed before and after modification indicate that the chemical changes did not significantly alter the surface morphologies of the new MOF.

### 3.6 Adsorption of Cu(II)

Copper (Cu) is an essential element for human metabolism, but excessive amounts of copper in drinking water can pose significant risks to human health. It is the second most hazardous element in drinking water after mercury (Hg). (Bolisetty et al., 2019). Elevated exposure to copper has been associated with various adverse effects on the body, including high blood pressure, increased respiration rate, and damage to the kidneys and liver. Symptoms of copper toxicity can include convulsions, cramps, vomiting, and in severe cases, even death (Zhu et al., 2020). The release of copper into water resources as a cationic metallic ion can exacerbate dangerous conditions. Prolonged exposure to copper-contaminated water can result in anemia, nausea, gastrointestinal discomfort, cyanosis, renal damage, and shortness of breath, potentially fatal consequences.

Eltaweil et al. (2021) developed UiO-66/GOCOOH@SA complex microbeads by combining UiO-66 and GOCOOH in SA microbeads. This composite material shows extraordinary potential for removing Cu(II) from wastewater. SEM images of GOCOOH revealed broken lamellae, suggesting disruption of the GO lamellae during carboxylation processes (Eltaweil et al., 2020). Dried SA beads exhibited a stretched form, and visible cracks and rough surfaces were observed due to dehydration (Elnashar et al., 2015). UiO-66/GOCOOH@SA composite beads consist of granular quasi-spherical UiO-66 combined with GOCOOH and SA. SEM images of the mixed beads revealed spherical shapes with highly rough surfaces, likely due to the different chemistry of the constituent parts. Notably, the UiO-66/GOCOOH@SA composite beads showed no cracks on the surface, indicating a superior organic structure compared to the SA beads. The adsorption capacity of UiO-66/GOCOOH@SA for Cu(II) was found to be 343.49 mg/g, and the adsorption rate remained above 87% even after five replicate experiments.

UiO-66, a specific subtype of MOFs, offers several distinct advantages over other MOFs in the adsorption of heavy metals. Its highly stable structure ensures durability and effectiveness in heavy metal ion adsorption processes, maintaining its adsorption capacity over prolonged periods. Secondly, UiO-66 has a large specific surface area, providing a higher density of active sites for enhanced adsorption efficiency and power than other MOFs. Furthermore, UiO-66 exhibits exceptional chemical stability over a wide range of pH conditions, enabling reliable adsorption performance even in acidic or alkaline environments, making it suitable for diverse environmental remediation applications. Moreover, the structure of UiO-66 can be easily modified or functionalized, allowing for enhanced selectivity and affinity towards specific heavy metal ions. Tailoring the functional groups within UiO-66 enables customization of its adsorption properties, leading to a more efficient and selective adsorption process.

In summary, the outstanding stability, high surface area, chemical resistance, and tunable functionality of UiO-66 make it a superior adsorbent for heavy metal ions compared to other MOFs. These unique properties highlight its potential in various environmental applications, including wastewater treatment and pollution remediation. The advantages of UiO-66 underscore its importance in heavy metal ion adsorption and emphasize its potential as a promising solution for environmental challenges associated with heavy metal contamination.

## 4 Adsorption parameters, conditions, and kinetic analysis of UiOs

### 4.1 Analysis of adsorption parameters

Understanding the effects of temperature, adsorbent dosage, adsorbent contact time, and pH on the adsorption process of UiO-66 is vital for optimizing adsorption efficiency and designing effective adsorption systems.

Temperature plays a significant role in adsorption, influencing kinetic energy and intermolecular interactions. Generally, higher temperatures reduce adsorption capacity due to increased thermal energy, which weakens the adsorbate-adsorbent interactions. Conversely, lower temperatures enhance adsorption efficiency by promoting stronger adsorbate-adsorbent binding.

The adsorbent dosage is an important parameter that affects the available adsorption sites. Increasing the adsorbent dosage provides more active sites for adsorption, leading to improved adsorption capacity. However, excessively high dosages may cause site blocking and hinder adsorption performance. Therefore, optimizing the adsorbent dosage is essential for achieving optimal adsorption efficiency.

Adsorbent contact time influences the rate of adsorption and equilibrium attainment. Prolonged contact time allows for more thorough interaction between the adsorbate and adsorbent, increasing adsorption capacity. Longer contact times will enable the adsorbate molecules to diffuse into the adsorbent pores, maximizing adsorption efficiency.

The initial concentration of the adsorbate affects the driving force for adsorption. Higher initial concentrations result in a more substantial concentration gradient, enhancing adsorption performance. However, there is an optimal range for the initial engagement, as excessively high concentrations can saturate the adsorbent and limit further adsorption.

Additionally, The pH of the solution plays a crucial role in the adsorption of heavy metal ions by adsorbents. It influences hazardous compounds’ surfaces, chemistry, distribution, and morphologies in aqueous systems. The pH controls the dissociation of acidic and basic substances in heavy metal ion solutions. When the pH exceeds a specific range, the adsorbents may undergo dissociation, which can limit their ability to adsorb heavy metal ions. The solubility and charge of the adsorbents are also affected by the pH of the solution. Therefore, it is essential to experiment and determine the optimal pH level for adsorption experiments.

The pH of the initial solution significantly affected the sorption capabilities of HP-UIO-66 and CNUIO for U(VI). For example, the sorption rate for U(VI) from CNUIO increased from 22.03% to 95.01% as the pH increased from 2 to 6. However, sorption decreased to 88.90% as the pH increased to 8. The starting pH also affected the removal efficiency of Hg(II) using UiO-66-NH_2_/AHMP, with maximum removal achieved at pH 6. The sorption capacity of Cu(II) increased as the pH increased from 3 to 5 and then remained relatively constant. Similarly, the adsorption capacity of Au(III) was highest at pH four and gradually decreased as the pH increased but sharply increased again with further pH increase.

In summary, the adsorption process of UiO-66 is influenced by several crucial parameters. Temperature affects the strength of adsorbate-adsorbent interactions, with higher temperatures generally reducing adsorption capacity. Adsorbent dosage plays a significant role in providing adsorption sites where an optimal dosage is required to achieve optimal performance. Longer adsorbent contact times allow for increased adsorption by facilitating more extensive interaction between the adsorbate and adsorbent. The adsorbate’s initial concentration impacts the adsorption’s driving force, with higher concentrations generally leading to improved adsorption performance within a specific range. The pH of the solution is an important parameter to consider in heavy metal ion adsorption studies, as it can significantly impact the adsorption capacity and behavior of the adsorbents.

### 4.2 Reusability of adsorbents

Reusability is critical when utilizing adsorbents to remove heavy metal ions from wastewater. It can effectively reduce the use of organic solvents required to generate the adsorbent, thus reducing the environmental pollution from organic solvents. In addition, reusability has a cost-saving economy. The study conducted by Yang et al. (Sun et al., 2016) demonstrated the excellent reusability of etched UiO-66/CTS, with a removal rate of 80.03% for Pb(II) and 75.34% for Cd(II) over five reuse cycles. Similarly, Wu et al. (2022) reported that CNUIO successfully removed U(VI) with a removal efficiency exceeding 85% in the first three experiments, indicating its potential for heavy metal ion absorption. However, the sorption rates gradually declined to approximately 80% after five iterations of desorption experiments. The results from five sorption-desorption studies indicated that CNUIO possessed a high capacity for short-term regeneration, but its ability weakened with increasing cycles.

### 4.3 Adsorption kinetic analysis

The kinetics of adsorption processes can be described using first-order and second-order kinetic models. The pseudo-first-order model (Eq. 1) and pseudo-second-order model (Eq. 2) are commonly used to analyze the kinetics of adsorption (Plazinski et al., 2009; Alberti et al., 2012; Tran et al., 2017).

$$\ln\left(q_{e}-q_{t}\right)=\ln q_{e}-K_{1}t$$ (1)

$$\frac{t}{q_{t}}=\frac{1}{K_{2}q_{e}^{2}}+\frac{t}{q_{e}}$$ (2)

where q_t_ (1/mg) is the amount of adsorbate adsorbed at a time t), q_e_ (mg/g) is the equilibrium adsorption capacity, and K_1_ (1/min) is the rate constant of the pseudo-first-order model, and K_2_ (g/mg-min) is the rate constant of the pseudo-second-order model.

Several studies have employed these kinetic models to analyze the adsorption of heavy metal ions using different adsorbents. To explain the kinetics of NH_2_-functionalized MOF adsorption for Cd(II) and Pb(II) at varied temperatures, Wang K. et al. (2017) used a postulated second-order model. According to the results, the recommended second-order model is well-conformed to the sorption values from Zhao et al. (2015). The sorption result shows that after 120 min of adsorption at 30°C, pH 6, and 10 mg/L concentration, 99.95% of the Pb(II) was removed, for the starting values at 40 mg/L, sorption of Cd(II) amounted to 177.35 mg/g. The Cd(II) sorption obtained 177.35 mg/g for the 40 mg/L starting values. Wu et al. (2019) analyzed the removal of metal ions [Eu(III), Hg(II), and Pb(II)] using UiO-66-EDTA and found that the fitted second-order model accurately simulated the kinetics of metal ion adsorption. Eltaweil et al. (2021) investigated the adsorption of MB and Cu(II) on UiO-66/GOCOOH@SA complex microbeads. They used both first- and second-order models and found that the pseudo-second-order model characterized the adsorption process well. Zhang et al. (2022) studied the sorption kinetics of a car amidoxime-modified UiO-66-(OH)_2_ derivative for U(VI). They compared the first-order and second-order kinetic models and found that the second-order model better fit the experimental data (*R* ^2^ = 0.9952 > *R* ^2^ = 0.9400).

In summary, the choice of kinetic model depends on the specific adsorption system and the experimental data. The second-order model is often found to provide a better fit for describing the kinetics of heavy metal ion adsorption.

## 5 Conclusion and outlook

Indeed, UiO materials, including UiO-66 and its composites, have shown good adsorption properties for heavy metal ions. The sorption processes observed in these studies indicate that the adsorption of heavy metal ions onto UiO materials follows chemisorption, which involves solid chemical interactions between the adsorbent and adsorbate. UiO materials have demonstrated several advantages as adsorbents, including short sorption times, good water stability, and high sorption capacities. These properties make them superior to other sorbents, as they can rapidly remove heavy metal ions from aqueous solutions while achieving high adsorption efficiencies.

When considering the selection of MOF materials for heavy metal ion adsorption, it is essential to consider factors such as the cost of production, environmental considerations associated with heavy metal ion adsorption, and the goal of achieving sustainable development. MOFs with favorable properties, such as UiO materials, hold promise in addressing these aspects and can potentially be effective and sustainable solutions for heavy metal ion removal in various applications. UiO-66, as a versatile MOF, has shown great potential for multiple applications, including the remediation of rich metal-contaminated environments. Its unique properties, such as high surface area, tunable pore size, and chemical stability, make it an attractive candidate for addressing environmental pollution challenges. In heavy metal ion removal, UiO-66 exhibits several advantages over other MOFs. Firstly, its exceptional adsorption capacity efficiently removes heavy metal ions from contaminated water sources. Studies have demonstrated that UiO-66 can achieve high sorption capacities for a wide range of heavy metal ions, including Pb(II), Cd(II), Cr(VI), and U(VI). This capability is crucial for effective environmental remediation.

Additionally, UiO-66 has shown excellent performance in terms of sorption kinetics. It exhibits fast adsorption rates, leading to shorter sorption times than other sorbents. Furthermore, the pH range of 4-8 has been identified as the optimal range for the adsorption studies using UiO materials. Within this pH range, UiO materials exhibit the best adsorption performance and the fastest adsorption rate. This suggests that the surface chemistry and functional groups of UiOs are highly responsive to the pH of the solution, leading to enhanced adsorption of heavy metal ions. This attribute is precious in practical applications where rapid removal of heavy metal ions is desired.

Moreover, UiO-66’s stability under varying environmental conditions enhances its suitability for real-world applications. Its robust structure and resistance to pH changes make it a reliable adsorbent for diverse environments. Furthermore, the reusability of UiO-66 adsorbents is a critical consideration. Researchers have explored various regeneration techniques, such as desorption with suitable solvents or pH adjustment, to restore the adsorption capacity of UiO-66 after each cycle. This capability promotes the sustainability and cost-effectiveness of UiO-66-based remediation processes.

UiO-66 exhibits excellent promise for applying heavy metal ion removal in environmental remediation. At present, MOFs for practical applications are still in the initial stage. The lack of synthetic methods for preparing MOFs in large quantities greatly hinders the industrialization of MOFs. Large-scale and low-cost green production methods are still under research. Most MOF-based materials are now available in powder form, limiting their use in practical situations. Although some MOFs have been industrialized, such as MOF WORX’s mass production of MOF powders using flow-through synthesis, this production method is limited to new MOF types, of which UiO-66 is one of the pristine MOFs. Synthesized powder MOFs are more challenging to handle and often require expensive and time-consuming techniques, such as centrifugation. They also pose inherent safety issues, such as respiratory health risks. This is very detrimental and limits MOF powders’ processing into valuable products. Therefore, further research and development are necessary to optimize their performance, scalability, and integration with existing remediation technologies. In conclusion, the existing green synthesis methods for large-scale preparation of MOF still need to be explored, and we still need to improve the synthesis methods for practical applications today with the help of the green synthesis methods for successful mass production of MOF in the laboratory, to promote the development of MOF industrialization.
